# Preoperative, intraoperative, and postoperative measures to further reduce spinal infections

**DOI:** 10.4103/2152-7806.76938

**Published:** 2011-02-21

**Authors:** Nancy E. Epstein

**Affiliations:** Clinical Professor of Neurological Surgery, The Albert Einstein College of Medicine, Bronx, NY 10467, and Chief of Neurosurgical Spine and Education, Winthrop University Hospital, Mineola, NY 11501, USA

**Keywords:** Antibiotic prophylaxis, infection, intraoperative prophylaxis, postoperative prophylaxis, preoperative prophylaxis, spinal surgery

## Abstract

**Background:**

The rate of postoperative spinal infections varies from 0.4% to 3.5%. Although the introduction of additional preoperative, intraoperative, and postoperative methods of prophylaxis should further reduce spinal infection rates, these measures will not succeed unless surgeons are well informed of their availability, utility, and efficacy. This study provides a review of several preoperative, intraoperative, and postoperative methods of prophylaxis that could minimize the risk of postoperative spinal infections. Various preoperative, intraoperative, and postoperative measures could further reduce the risk of spinal infections. Preoperative prophylaxis against methicillin-resistant *Staphylococcus aureus* could utilize (1) nasal cultures and Bactroban ointment (mupirocin), and (2) multiple prophylactic preoperative applications of chlorhexidine gluconate (CHG) 4% to the skin. Intraoperative prophylactic measures should not only include the routine use of an antibiotic administered within 60 min of the incision, but should also include copious intraoperative irrigation [normal saline (NS) and/or NS with an antibiotic]. Intraoperatively, instrumentation coated with antibiotics, and/or the topical application of antibiotics may further reduce the infection risk. Whether postoperative infections are reduced with the continued use of antibiotic prophylaxis remains controversial. Other postoperative measures may include utilization of a silver (AgNO_3_)-impregnated dressing (Silverlon dressing) and the continued use of bed baths with CHG 4%. The introduction of multiple preoperative, intraoperative, and postoperative modalities in addition to standardized prophylaxis may further contribute to reducing postoperative spinal infections.

## INTRODUCTION

Multiple available preoperative, intraoperative, and postoperative methods of prophylaxis may be utilized to prevent spinal infections. Although most focus on the immediate preoperative administration of antibiotic prophylaxis within 1 h prior to surgery, little attention has been paid to other adjunctive measures that may also reduce the risk of postoperative spinal infections. Preoperative measures to reduce methicillin-resistant *Staphylococcus aureus* (MRSA) rates include the use of nasal cultures and Bactroban ointment (mupirocin), chlorhexidine gluconate (CHG) 4% showers/baths prior to surgery, and the utilization of clippers to remove hair rather than using razors. Perioperative measures include the timely administration of prophylactic antibiotics, the use of copious intraoperative irrigation with normal saline (NS), the implantation of antibiotic-impregnated devices, and the local application of antibiotics. Postoperative measures reaching beyond the disputed duration of antibiotic prophylaxis, may also include silver-impregnated dressings (AgNO_3_), and continued bathing with CHG 4%.

Although surgeons may adopt several of the adjunctive measures mentioned, the 0.4%–3.5% incidence of infections, more frequently seen with the use of spinal instrumentation, further thought should be given to basics: (1) sterile technique, (2) avoiding talking during surgery to reduce the bacterial count, (3) the meticulous handling of tissues and hemostasis, and the placement of drains[8,10,19] [Table 1].

**Table 1:** Preoperative and postoperative measures to reduce spinal infections

Variable	Measure
Preoperative nasal swab	Mupirocin (Bactroban)
	Apply intranasal b.i.d. ×7 days
Preoperative patient bathing	Chlorhexidine gluconate
	Baths b.i.d. ×7–14 days
	Night before/morning of surgery
Preoperative hair cutting	Electric clippers
	Avoid razors
Preoperative surgical scrub	Chlorhexidine gluconate brushes
	Avagard
Preoperative skin preparation	Alcohol alone
	Alcohol with 4% chlorhexidine gluconate
Preoperative prophylactic antibiotic	Ancef 2 g (not penicillin allergic)
Within 1 h of surgery	Vancomycin 1 g (penicillin allergic)
Postoperative antibiotic prophylaxis	
Preoperative dose alone	1 Preoperative dose
Postoperatively (controversial)	Continued (regimen)
Intraoperative irrigation	Normal saline alone (2000 cc/h)
Throughout surgery/high volume	Normal saline with antibiotic
Intraoperative local antibiotic	Antibiotic-coated spheres
Antibiotic-coated instrumentation	Cationic antimicrobial peptides
	On instrumentation (impregnated)
Postoperative silver dressing	Applied postoperatively
	Used for 7–14 postoperative days
Postoperative bathing	Continued bed baths b.i.d.
	4% Chlorhexidine gluconate
C-reactive protein levels	Preoperative baseline
	Postoperative studies
	1, 3, 5, 7 days postoperatively

This commentary, therefore, allows surgeons to go beyond the utilization of preoperative prophylactic antibiotics to prevent infections, or the necessity for posteroperative antibiotics to treat infections.

### Nasal Colonization with Methicillin Resistant

#### Staphylococcus aureus

The incidence of postoperative spinal infections with MRSA could be reduced by the preoperative culturing of patients for intranasal MRSA. One study evaluated multiple series of preoperative patients who tested positive for intranasal MRSA, and were treated prior to surgery with mupirocin (Bactroban: typically utilized b.i.d. for 7 days).[15] This regimen was accompanied by the application of a 2% CHG preparation on the skin. The overall number of surgical infections was decreased by 63%; those due to MRSA were diminished by 78%. Interestingly, this reduction in postoperative infections saved 2 small hospitals $240,000.

In another study, the institution of a universal screening program included the use of nasal swabs and administration of mupirocin and chlorhexidine showers for carries of MRSA and methicillin-sensitive *Staphylococcus aureus* (MSSA).[13] The study included 7019 patients; 1588 or 22.6% proved to be *Staphylococcus aureus* carriers. Of these, 309 or 4.4% were MRSA carriers. A significantly greater number of infections occurred for MRSA carriers, but no statistically significant increase in the rate of infection occurred for those with MSSA vs noncarriers. The overall infection rate for screened patients was 13/7019 (0.19%); this was significantly lower than that noted without such screening 24/5293 (0.45%). The authors concluded that prescreening and treating MRSA and MSSA carriers might reduce the risk of postsurgical orthopedic infections.

Finally, in a randomized, double-blinded, placebo-controlled multicenter trial, the frequencies of MSSA nasal carriers were identified with a real-time polymerase chain reaction (PCR) assay.[1] Positive studies were followed by the administration of mupirocin nasal ointment and chlorhexidine soap washes to the skin. The series included nasal swabs positive for MSSA from 1251 of 6771 (18.5%) patients. Out of 917 patients enrolled, 808 (88.1%) had surgery; the rate of infection for MSSA was 3.45% (17/504 patients) compared with 7.7% (32/413 patients) in the placebo group.

Although the author has recently asked that most preoperative spinal patients utilize mupirocin nasal ointment the week prior to surgery, other surgeons may choose to use it in the presence of certain “risk factors for MRSA.” This includes patients with prior histories of surgery and/or other MRSA infections; they should not only utilize mupirocin prior to spinal surgery, but may benefit from a consultation from an infectious disease expert. Additionally, patients with cats/fish/other animals should also take this nasal ointment, as several of these animals are noted carriers of MRSA. There appears to be a minimal “down-side” (morbidity) for utilizing intranasal mupirocin, and most patients are compliant.

### Risk Factors That Increase Operative Risk (General)

Complex protocols should be utilized to decrease the risk of infection following any type of surgery, particularly in patients with multiple comorbidities. In a cardiac surgery study, a multi-interdisciplinary approach was adopted to control/decrease the frequency of deep sternal infections.[5] Preventive measures included MRSA nasal screening prior to surgery, hair clipping rather than shaving, changing surgical gloves following sternotomy/after wiring, and not changing the original dressing for 48 h postoperatively. The authors concluded that the following risk factors correlated with a significant increased risk of infection; age over 68 years, diabetes, and higher intraoperative blood glucose levels. In another series of patients undergoing spinal surgery, the difference in infection rates for outpatient (1%) vs inpatient (2.8%) procedures was largely attributed to obesity [increased body mass index (BMI); more obese patients typically had surgery as inpatients].[22] Furthermore, all 7 patients undergoing readmission for postoperative infection were obese (BMI ≥ 30), and exhibited more comorbidities.

Presently, few surgeons are actively involved in creating strict preoperative, intraoperative, and postoperative protocols which should be adjusted according to different patients’ comorbid factors. Perhaps greater attention to these comorbid variables prior to surgery (eg, diabetes, obesity, peripheral vascular/coronary artery/pulmonary disease) would not only allow various protocols to be enacted, but may also influence the surgeon as to what he/she chooses to implant. It is certainly, well known that diabetics have a higher risk of infection and pseudarthrosis. In these patients, shorter procedures with well-controlled intraoperative and postoperative blood glucose levels, and limited/absent instrumentation may reduce the risk of infection.

### Preoperative Hand Scrub Alternatives for Surgeons

Multiple new scrub techniques and products have been proposed for surgeons. One of these, Avagard (Cardinal Health, East Rutherford, NJ, USA), is a new waterless, scrubless, and brushless hand antiseptic that the Food and Drug Administration has approved to replace scrub brushes prior to surgery.^[25]^ In one series, Avagard was utilized as a preoperative hand/scrub preparation for urological surgeons. The operative infection rates were compared for 1800 cases prospectively performed by surgeons utilizing Avagard vs 1800 previous consecutive procedures performed utilizing traditional scrub brushes. The wound infection rate for Avagard was 2/1800 (0.11%) vs 3/1800 (0.17%) for those using the hand brushes. These results documented no significant difference between the two. Notably, all infections were managed with one course of oral antibiotic therapy, without further complications, and the hand scrub brushes were twice the cost of Avagard, with longer scrub time using the brushes.

Prior to all spinal cases, I have personally chosen to first scrub with Avagard, and follow this with the full brush technique. For any successive cases, I still would utilize both the Avagard and brushes.

### Alternatives for Preoperative Skin Preparation

Minimizing the number of skin organisms utilizing preoperative skin antiseptic techniques should prove valuable in reducing postoperative infection rates. One study compared the relative efficacy of the preoperative preparation of chlorhexidine–alcohol (CA) vs povidone–iodine (PI).[2] Adults undergoing surgery in 6 hospitals had preoperative skin preparation performed with one or the other product. The rate of surgical site infections, including the types of infections, were evaluated within 30 days of the surgery. Out of 849 patients, the overall rate of surgical site infection was reduced to 9.5% for the CA groups (409 patients) vs 16.1% for the PI group (440 patients). Furthermore, CA proved more effective against both superficial (4.2% CA vs 8.6% for PI) and deep infections (CA 1% vs PI 3%) following clean/contaminated surgical procedures.

There are also different alternatives for skin preparation prior to the percutaneous implantation of devices (pacemakers, endovascular foreign bodies). Although the more common preparations are the water-soluble iodophors or CHG, alcohol-based solutions in one series proved to be “quick, sustained, and durable, with broader spectrum antimicrobial therapy.”[14] When prosthetic devices were implanted, alcohol best reduced skin colony counts, thereby contributing to a greater reduction in the rates of infected instrumentation.

In another study, multiple factors were introduced to reduce the 30% infection rate (1 million cases) that followed cesarean sections (4 million births/year).[18] Prospectively, the authors introduced a full staff education program, preoperative skin preparation using CHG (no rinse cloths), CHG with alcohol for intraoperative skin preparation, and a modified program for instrumentation sterilization. Combined, these measures significantly reduced the postoperative infection rate from 7.5% to 1.2%.

In a study involving total joint procedures, skin prophylaxis utilized 2% CHG no rinse cloths the night before surgery and the morning of surgery; with this CHG protocol, the infection rates were reduced from 3.19% to 1.59%.[3]

The patients are now asked to bathe twice a day and utilize CHG during the shower or bath, taking care to exclude the eyes, ears, and groin. Asking patients to bathe twice helps ensure that they bathe at least once per day, an important factor particularly in the older population. There have been few complaints, and it allows the patients to become active contributors, along with their spouses/ other family members, to their own safety/care.

## INTRAOPERATIVE IRRIGATION

There has been considerable discussion regarding whether intraoperative irrigation reduces the risk of infection, and what types of intraoperative irrigation (e.g. normal saline (NS) with/without antibiotics) are the best. In one study, involving children with perforated appendices, patients were managed prior to closure utilizing 1–2 L of NS wound irrigation without antibiotics.[20] There were 47 simple perforations, and 22 had complicated perforations. Four (5.8%) complications followed surgery; 2 small seromas that resolved, 1 patient with adhesions/obstruction and an enterocutaneous fistula requiring surgical decompression, and 1 with a wound infection. The authors concluded that vigorous irrigation and a subcuticular skin closure decreased the postoperative infection rate.

Another study utilized 3 different regimens with/without intraoperative irrigation to reduce the incidence of ventriculoperitoneal shunt infections.[6] Group A patients did not have any irrigation, Group B patients received saline with Amikacin, whereas Group C patients received NS irrigation alone. A total of 150 shunts were performed in all 3 groups; 61 patients (A), 40 patients (B), 49 patients (C). Nine patients developed infections within 90 (6%) postoperative days. Eight (13.3%) infections occurred in patients where no irrigation was utilized. Alternatively, for groups B and C, the combined infection rate for the use of copious irrigation was only 1.1%; 0% for Group B, and 1 (2%) for Group C.

A further study sought to reduce spinal infections by utilizing saline irrigation in 223 consecutive cases.[24] The frequency of surgical infection was 6.3%. The average volume of NS used for irrigation was less for the infected vs noninfected patients (the latter average of >2000 mL/h). The incidence of infection positively correlated with longer operations (>3 h), delayed operations following trauma, diabetes, and greater blood loss (>300 cc); there was no correlation with older age, BMI, or length of hospital stay prior to surgery.

Prior to writing this review, I would irrigate every 15 min during the case with Bacitracin/Polymyxin-B sulfate, and utilize a liter of this irrigant with a pulse-evacuator at the end of the procedure. After having reviewed the literature, which focuses on the attributes of NS alone utilized at very high volumes, I have tripled (full bulb syringe ×3) the volume of antibiotic impregnated irrigation fluid utilized every 15 min.

### Intraoperative Implanted Antibiotics or Antibiotic-Coated Devices

There has been interest in whether the local addition of antibiotics or antibiotic-coated instrumentation would reduce postoperative spinal infection rates. In an animal model (rabbit), *Staphylococcus aureus* and local antibiotics were administered. Gentamicin was administered via “controlled-release” microspheres [LGA (polylactide–;coglycolide–nanoparticles)], which yielded an adequate local concentration for up to 7 postoperative days.[21] The rabbits were also given a systemic cephalosporin along with antibiotic-impregnated rods. *S. aureus* was applied with/without gentamicin LGA microspheres. Surgical sites became infected in 75% of control sites, but gentamicin microspheres reduced the infection rate by 50%.

Another study utilized loading/local use of cationic antimicrobial peptides on implant surfaces to reduce surgical site infections.[12] A thin layer of microporous calcium phosphate coating was utilized in addition to a broad-spectrum antibiotic on the implant (AMP Tet 213); the combination proved to be effective against gram-positive and gram-negative bacteria.

As yet, I have not adopted the use of antibiotic-impregnated devices/instrumentation, nor have I locally applied antibiotic powder into the wound itself. As more literature becomes available, and more antibiotic-impregnated devices are put on the market, both measures might become useful.

### Optimal Choice and Timing of Antibiotic for Prophylaxis

To better determine whether prophylactic antibiotics reduce the risk of intradiscal infection during spinal surgery, the blood and intradiscal levels of cephazolin were assessed in 30 (average age 42 years) patients having lumbar spinal fusions.[23] Patients received 1 g of cephazolin during 1–2 level lumbar fusions. The minimal inhibitory concentration against *S. aureus* was considered to be 1 mg/L after the intravenous administration of this drug. Intravenous blood samples were obtained prior to surgery and during disc removal. The highest antibiotic level in the serum (31.1–148 mg/L) was achieved at 7 min, whereas in the disc (0–9.5 mg/L), it occurred between 37 and 53 min following intravenous injection. Although all serum levels were over the critical 1-mg/L level, only half had the 1-mg/L minimal inhibitory concentration level in the disc material itself.

The Surgical Care Improvement Project (SCIP) over a 2-year period retrospectively looked at 6 variables to reduce postoperative complications, including surgical site infection due to the timely administration of preoperative antibiotics.[17] Compliance with SCIP improved from 80% to 94% over 2 years. Improved antibiotic dosing times improved to 90% compliance, whereas the appropriate choice of the antibiotic improved to 100% compliance. Nevertheless, stopping antibiotics within 24 h of surgery did not succeed due to differing opinions/variables.

Surgical infections have been reduced by the administration of preoperative antibiotics within 60 min of surgery (incision).[16] These authors added an “oral antibiotic verification” to the routine preoperative time out (patient identification, operation, and surgical site verification) in order to determine whether they could increase compliance with this antibiotic regimen. Timely administration of antibiotics was defined as ≤60 min, early was >60 min prior, and late following incision or not at all. Of 715 cases analyzed, 315 did not require treatment, 88 were inpatient procedures, and in 22 the case records were not complete. Therefore, prior to the intervention, 87/97 or 90% compliance was seen with timely antibiotic administration. Postintervention, the compliance was 163/193 cases or 85%. There was no significant difference noted in compliance with/without the use of the preoperative time out for prophylactic antibiotic administration.

For spinal cases, I typically use 2 g of Ancef (Cefuroxime) administered intravenous push within 15–30 min of the incision. For patients who are penicillin allergic, vancomycin 1 g is given slowly intravenously over 60 min. In addition to the Ancef, 80 mg of gentamicin is given over 60 min before surgery, in order to add its impact against MRSA.

### Silverlon Dressings to Prevent Postoperative Infections

Silverlon, which contains the organic salt AgNO_3_, has been used as a topical agent in medicine for centuries. In 1968, silver sulfadiazine became available and was utilized to facilitate wound healing, particularly in the treatment of burns. More recently, various slow-release silver dressings have become available, including (Acticoat; Smith and Nephew, Largo, FL, USA), Silverlon (Argentum, Lakemont, GA, USA), and Silvasorb (Medline Industries, Inc., Mundelein, IL, USA) to reduce postoperative infections.[7] The first study evaluated the efficacy of weekly dressing changes containing AgNO_3_ to reduce infection and contain cost (eg, avoiding daily or more frequent dressing changes) and involved Sprague–Dawley rats that received a burn.[7] On day 3, the wound was infected with *Pseudomonas aeruginosa* and *S. aureus*. There were 4 treatment groups: untreated control, Acticoat, Silvasorb, and Silverlon. Dressings remained on wounds for 10 days. All 3 silver dressing products proved comparable and superior to no silver impregnated dressings. Additionally, dressing changes on a weekly basis proved to be feasible, both economically and medically.

The incidences of superficial vs deep infection rates were compared utilizing Silverlon dressings (SD: Silverlon; Argentum Medical, LLC, Lakefront, GA, USA) vs routine dressings (RD: gauze, alcohol swab) for 2 weeks following lumbar spinal surgery.[4] The initial 128 patients received RD, whereas the second group of 106 patients received SD. Three of the 128 patients undergoing multilevel laminectomies with instrumented fusions with RD developed deep infections, all managed with 6 weeks of antibiotic therapy; no second operations were needed. Additionally, 11 of 128 had superficial infections with RD; 7 were placed on oral therapy alone for 7–10 days, whereas 4 were referred to plastic surgeons for superficial wound revision. In 106 SD patients, there were no deep or superficial infections.

I utilize Silverlon dressings (Argentum Medical, LLC, Lakefront, GA) on spinal wounds when staples rather than steri-strips are applied (typically only anterior cervical, anterior iliac crest). These dressings are removed and reapplied daily for each of 7 days after being washed with sterile water. Notably, the silver-impregnated side is applied directly to the skin, and is followed by the application of sterile gauze and tape. If, however, the dressing is soiled/dropped/contaminated, it should be replaced with a new one. The use of these dressings has effectively reduced superficial wound infections, and the need for a home health aid to assess the patients’ wounds at home has been, perhaps, the most beneficial impact derived from using this dressing.

### Incidence of Postoperative Spinal Infections

The incidence of postoperative spinal infections varies from 0.4% to 3.5%.[8,10,19] One study included 27 (2.7%) of 997 spinal procedures that became infected.[9] Risk factors predisposing toward infections that were predominantly attributed to coagulase-negative staphylococci included older age (mean 59 years), diabetes, longer hospital stays, and the implantation of a foreign body.

In another series, the rate of infection during spinal surgery was evaluated in 1597 consecutive patients receiving a single-dose vs multiple-dose antibiotic prophylaxis for varied lumbar surgical procedures (discs, degenerative spondylolisthesis, spinal stenosis, reoperations, and so on).[10] There were 1133 patients who received multiple doses of antibiotics vs 464 in the single-dose populations. The frequency of instrumented fusions was comparable in the multiple 43% vs single-dose groups 39%. The rate of infection was 0.8% for the multiple dose group and 0.4% for the single dose groups; this difference was not significant. Interestingly, in the multidose group 5 of 6 organisms were resistant strains, whereas no such strains were identified in the single-dose populations.

In another study, in 284 patients undergoing noninstrumented spinal procedures, half received no postoperative antibiotics, while the other half did.[8] The combined incidence of spinal infection was 2.1% (6/284). However, the frequency of infection was a higher 2.8% (4/141) for those receiving postoperative antibiotics compared with the 1.4% (2/143) who did not. This difference was not statistically significant and 2 patients who received prolonged antibiotics developed pseudomembranous colitis.

Although the ideal postoperative treatment of infection following an instrumented fusion may be instrumentation removal, this may not be feasible. Alternatives to the removal of instrumentation may include the prolonged use of antibiotic therapy and wound irrigation/debridement.[19] In a series of infections occurring following 737 thoracolumbar spinal instrumentation procedures, 26 (3.5%) infections occurred; 19 were early, while 7 were delayed. Seventeen of 19 early infections were effectively treated with prolonged antibiotic use consisting of 4–6 weeks of intravenous antibiotics followed by an added 4–12 week course of oral therapy. Alternatively, 6 of 7 with delayed infections ultimately required instrumentation removal to eradicate the infection.

One major issue we have to deal with at present is whether we actually accept an infection rate that ranges from 0.4% to 3.5%.[8,10,19] Prior to the advent of antibiotics, it is likely that more meticulous sterile technique minimized the risk of acquiring an intraoperative infection. However, even now with the availability of antibiotics for prophylaxis and for treating spinal infections, greater attention should once again be paid to the “basics”: the preparation and draping of the patient, avoiding talking at the operating room table, limit the operative time, carefully handling tissue with instruments and not gloved hands, changing gloves frequently, keeping operating room doors closed, limiting traffic, stringently observing new personnel for sterile technique, and minimizing the number of breaks/change in personnel for scrub technicians, and other personnel are just some of the factors involved in limiting the risks of infection.

Another major consideration is whether in fact the patient requires instrumentation. Specifically, in the lumbar spine in older patients, those with osteoporosis, diabetes, or other “relevant” comorbid factors, there may be instances in which decompressions alone or with a noninstrumented (in situ) fusion rather than an instrumented fusion may be “more” appropriate. Eliminating the foreign body effectively limits both the operative time and the increased risk of infection correlated with organisms adhering to “hardware.” Furthermore, if an infection arises, there will be less need for a second operation, as there is no instrumentation to be removed. In geriatric patients, therefore, I would typically perform either no fusion or in the presence of degenerative spondylolisthesis, a noninstrumented fusion, but only rarely would add instrumentation. The short- and long-term results for the majority of these noninstrumented fusions have proved to be quite adequate.

## EARLY DIAGNOSIS OF POSTOPERATIVE INFECTION

C-reactive protein (CRP) levels in the serum may increase within 6 h following the initiation of a bacterial infection, and may be utilized to diagnose and follow the course of and treatment of infections.[11] Normally after surgery, the CRP rapidly increases, and then gradually decreases to normal levels. In this series, CRP levels were utilized to detect early infections in 348 consecutive spinal procedures. Blood tests were obtained preoperatively and on postoperative days 1, 3, and 5 for single-level procedures, and day 7 for more extensive operations. All patients received a prophylactic antibiotic. CRP normally increased and then decreased in 332/348 patients (95.4%). The CRP was typically 14.9 on day 1, 15.4 on day 3, and 7.9 on day 5. In cases where the CRP continued to rise after postoperative days 5–7, a new antibiotic was used for the presumptive infection. There were 16 (4.6%) infections; the second rise occurred in 12 cases with a steady rise in 4 of CRP.

If there is a question of a postoperative infection, the use of successive erythrocyte sedimentation rate (ESR) and CRP studies has proven useful along with enhanced magnetic resonance imaging scans. Although ESR and CRP studies will typically demonstrate greater sensitivity to the effectiveness of antibiotic/other therapy, MR results tend to “lag,” demonstrating a slower “resolution” of pathological findings.

## CONCLUSION

Multiple adjunctive measures may be utilized to try to limit devastating postoperative spinal infections. These include preoperative nasal cultures for MRSA and the use of prophylactic mupirocin plus preoperative bathing with CHG. Preoperative comorbid factors, particularly diabetes, increase the postoperative infection rate, as does the presence of morbid obesity. The preoperative utilization of prophylactic antibiotics supplemented with copious intraoperative irrigation with NS alone with/without antibiotics helps reduce the risk of postoperative spinal infections. Following surgery, there is no substantive data that show that continued antibiotic use reduces the postoperative infection rate, although there is a “trend” toward more resistant organisms occurring. The use of silver-impregnated dressings appears to reduce postoperative infection, which may also be detected early by following the patients’ postoperative CRPs.

Each surgeon may choose to adopt one or more of the adjuncts mentioned to limit infections following spine surgery. Critical to the limitation/elimination of infection, is the heightened awareness associated with the adoption of formal protocols, which alone may help decrease the infection rate.

## References

[1] Bode LG, Kluytmans JA, Wertheim HF, Bogaers D, Vandenbroucke-Grauls CM, Roosendaal R (2010). Preventing surgical-site infections in nasal carriers of *Staphylococcus aureus*. N Engl J Med.

[2] Darouiche RO, Wall MJ, Itani KM, Otterson MF, Webb AL, Carrick MM (2009). Chlorhexidine-Alcohol versus Povidone-Iodine for Surgical-Site Antisepsis, N Engl J Med 2010;362:18-28.Eiselt D. Presurgical skin preparation with a novel 2% chlorhexidine gluconate cloth reduces rates of surgical site infection in orthopaedic surgical patients. Orthop Nurs.

[3] Epstein NE (2007). Do silver-impregnated dressings limit infections after lumbar laminectomy with instrumented fusion?. Surg Neurol.

[4] Graf K, Sohr D, Haverich A, Kuhn C, Gastmeier P, Chaberny IF (2009). Decrease of deep sternal surgical site infection rates after cardiac surgery by a comprehensive infection control program. Interact Cardiovasc Thorac Surg.

[5] Hayashi T, Shirane R, Yokosawa M, Kimiwada T, Tominaga T (2010). Efficacy of intraoperative irrigation with saline for preventing shunt infection. J Neurosurg Pediatr.

[6] Heggers J, Goodheart RE, Washintron J, McCoy L, Carino E, Dang T (2005). Therapeutic efficacy of three silver dressings in an infected animal model. J Burn Care Rehabil.

[7] Kakimaru H, Kono M, Matsusaki M, Iwata A, Uchio Y (2010). Postoperative antimicrobial prophylaxis following spinal decompression surgery: Is it necessary?. J Orthop Sci.

[8] Kanafani ZA, Dakdouki GH, El-Dbouni O, Bawwab T, Kanj SS (2006). Surgical site infections following spinal surgery at a tertiary care center in Lebanon: Incidence, microbiology, and risk factors. Scand J Infect Dis.

[9] Kanayama M, Hashimoto T, Shigenobu K, Oha F, Togawa D (2007). Effective prevention of surgical site infection using a Centers for Disease Control Prevention guideline-based antimicrobial prophylaxis in lumbar spine surgery. J Neurosurg Spine.

[10] Kang BU, Lee SH, Ahn Y, Choi WC, Choi YG (2010). Surgical site infection in spinal surgery: Detection and management based on serial C-reactive protein measurements. J Neurosurg Spine.

[11] Kazemzadeh-Narvat M, Kindrachuk J, Duan K, Jenssen H, Hancock RE, Wang R (2010). Antimicrobial peptides on calcium phosphate-coated titanium for the prevention of implant associated infections. Biomaterials.

[12] Kim DH, Spencer M, Davidson SM, Li L, Shaw JD, Gulczynski D (2010). Institutional prescreening for detection and eradication of methicillin-resistant *Staphylococcus aureus* in patients undergoing elective orthopaedic surgery. J Bone Joint Surg Am.

[13] Lepor NE, Madyoon H (2009). Antiseptic skin agents for percutaneous procedures. Rev Cardiovasc Med.

[14] Lipke VL, Hyott AS (2010). Reducing surgical site infections by bundling multiple risk reduction strategies and active surveillance. AORN J.

[15] Nemeth TA, Beilman GH, Hamlin CL, Chipman JG (2010). Preoperative verification of timely antimicrobial prophylaxis does not improve compliance with guidelines. Surg Infect (Larchmt).

[16] Potenza B, Deligencia M, Estigoy B, Faraday E, Snyder A, Angle N (2009). Lessons learned from the institution of the Surgical Care Improvement Project at a teaching medical center. Am J Surg.

[17] Rauk PN (2010). Educational intervention, revised instrument sterilization methods, and comprehensive preoperative skin preparation protocol reduces cesarean section surgical site infections. Am J Infect Control.

[18] Sierra-Hoffman M, Jinadatha C, Carpenter JL, Rahm M (2010). Postoperative instrumented spine infections: A retrospective review. South Med J.

[19] Sookpotaroam P, Khampiwmar W, Termwattanaphakdee T (2010). Vigorous wound irrigation followed by subcuticular skin closure in children with perforated appendicitis. J Med Assoc Thai.

[20] Stall AC, Becker E, Ludwig SC, Gelb D, Poelstra KA (2009). Reduction of postoperative spinal implant infection using gentamicin microspheres. Spine.

[21] Walid MS, Robinson JS, Robinson ER, Brannick BB, Aijan M, Robinson JS (2010). Comparison of outpatient and inpatient spine surgery patients with regards to obesity, comorbidities, and readmission for infection. J Clin Neurosci.

[22] Walters R, Moore R, Fraser R (2006). Penetration of cephazolin in human lumbar intervertebral disc. Spine.

[23] Watanabe M, Sakai D, Matsuyama D, Yamamoto Y, Sato M, Mochida J (2010). Risk factors for surgical site infection following spine surgery: Efficacy of intraoperative saline irrigation. J Neurosurg Spine.

[24] Weight CJ, Lee MC, Palmer JS (2010). Avagard hand antisepsis vs. traditional scrub in 3600 pediatric urologic procedures. Urology.

